# Role of Long Noncoding RNAs in Smoking-Induced Lung Cancer: An In Silico Study

**DOI:** 10.1155/2022/7169353

**Published:** 2022-04-30

**Authors:** Lei Ge, Shishun Zhao, Jianguo Sun, Jianhua Cheng, Shaokun Wang

**Affiliations:** ^1^Center for Applied Statistical Research, School of Mathematics, Jilin University, Changchun 130012, China; ^2^Department of Statistics, University of Missouri, Columbia, MO 65211, USA; ^3^Department of Emergency, The First Hospital of Jilin University, Changchun 130012, China

## Abstract

The prevalence of lung cancer induced by cigarette smoking has increased over time. Long noncoding (lnc) RNAs, regulatory factors that play a role in human diseases, are commonly dysregulated in lung cancer. Cigarette smoking is closely related to changes in lncRNA expression, which can affect lung cancer. Herein, we assess the mechanism of lung cancer initiation induced by smoking. To calculate the impact of smoking on the survival of patients with lung cancer, we extracted data from The Cancer Genome Atlas and Gene Expression Omnibus databases and identified the differentially expressed genes in the lung cancer tissue compared to the normal lung tissue. Genes positively and negatively associated with smoking were identified. Gene Ontology, Kyoto Encyclopedia of Genes and Genomes, and Cytoscape analyses were performed to determine the function of the genes and the effects of smoking on the immune microenvironment. lncRNAs corresponding to smoking-associated genes were identified, and a smoking-related lncRNA model was constructed using univariate and multivariate Cox analyses. This model was used to assess the survival of and potential risk in patients who smoked. During screening, 562 differentially expressed genes were identified, and we elucidated that smoking affected the survival of patients 4.5 years after the diagnosis of lung cancer. Furthermore, genes negatively associated with smoking were closely associated with immunity. Twelve immune cell types were also found to infiltrate differentially in smokers and nonsmokers. Thus, the smoking-associated lncRNA model is a good predictor of survival and risk in smokers and may be used as an independent prognostic factor for lung cancer.

## 1. Introduction

Lung cancer, one of the most frequent malignant neoplasms, is a leading cause of cancer-related death, with an estimated 1.8 million deaths reported globally in 2020 [1]. The 5-year survival rate for advanced late-stage lung cancer remains poor at approximately 5% [2, 3]. Furthermore, lung cancer has been reported to have the worst prognosis among all cancers [4]. Survival remains poor because most patients are diagnosed at an advanced stage [5]. Prior epidemiological studies have identified smoking as an important cause of lung cancer [6, 7]. Notably, among the 4,000 chemicals identified in cigarette smoke, more than 60 are established carcinogens, according to the assessments performed by the International Agency for Research on Cancer [8]. These chemical carcinogens are the cause of cancer from cigarette smoke [9]. Challenges exist in predicting smoking-induced lung cancer, and previous studies on the relationship between smoking and lung cancer have yielded mixed results, with some suggesting a close association [10, 11], whereas others report no relationship [12]. Kadara et al. provide information on the early events in the molecular pathogenesis of lung cancer [13], but to improve prognosis, the molecular pathogenesis of smoking-induced lung cancer must be further studied.

Smoking is associated with changes in the expression of many long noncoding RNAs (lncRNAs) [14], which are non-protein-coding transcripts that span more than 200 nucleotides in length and are closely related to a variety of physiological functions [15]. The dysregulation of lncRNA expression has been noted in diseases [16, 17]. It has been reported that the abnormal expression of lncRNA is directly related to lung cancer [18, 19]. Thus, lncRNAs that are related to smoking and accurately predict the prognosis of lung cancer need to be identified through screening and comparison.

In this study, we divided differentially expressed smoking-related genes into positively and negatively associated genes and analyzed their functions to better understand their role in the development of lung cancer. The discovery of a relationship between negatively associated genes and immunity would provide theoretical support for the study of the immune microenvironment in smoking-induced lung cancer. We therefore obtained the lncRNAs corresponding to the positively and negatively associated genes through screening and built a model to compare the accuracy of the negative and positive lncRNA models in predicting the prognosis of lung cancer. Additionally, we compared the lncRNAs proposed in the previous reports. We expect our findings to improve our understanding of the mechanisms by which smoking induces lung cancer and that they will aid the development of a better prognostic factor for lung carcinogenesis.

## 2. Materials and Methods

### 2.1. Raw Data

The lung cancer-related RNA sequencing and clinical data used in this study were obtained from The Cancer Genome Atlas (TCGA; https://portal.gdc.cancer.gov/; 535 tumor samples and 59 normal samples) and Gene Expression Omnibus (GEO; gse68465; 442 tumor samples and 20 normal samples) databases.

### 2.2. Identification of Differentially Expressed Genes

The limma and edgeR packages in R (The R Foundation for Statistical Computing, Vienna, Austria) were used to analyze lung cancer samples in the two databases. By selecting logFCfilter = 1 and fdrFilter = 0.05 as the filter conditions, genes differentially expressed between lung cancer and normal lung tissues were screened from the two databases. Graphs were drawn using PheatMap and the ggplot2 package.

### 2.3. Weighted Correlation Network Analysis (WGCNA)

Differentially expressed genes in the two databases were analyzed using the WGCNA package (https://www.genetics.ucla.edu/labs/horvath/CoexpressionNetwork/Rpackages/WGCNA/) [20]. The gene expression data were used to construct a scale-free network. To ensure the reliability of the network, the samples with very low gene expression were excluded. The appropriate soft threshold was determined according to the standard scale-free network (soft threshold = 5), and the power function was used to calculate the correlation between different genes. After determining the soft threshold, we used the WGCNA algorithm to build the module.

### 2.4. Sample Subgrouping for Correlation with Smoking

Using WGCNA, the modules closely related to smoking and genes in the modules were obtained. The Venn package was used to retrieve the intersection of the differential genes in the two datasets and the smoking-associated genes in the two databases. Genes that were positively and negatively associated with smoking were obtained. The expression levels of genes and lncRNAs were compared using the limma package in R, selecting the conditions CorFilter = 0.4 and PValueFilter = 0.01 for filtering. lncRNAs positively and negatively associated with smoking were then selected. The samples were then divided into high- and low-risk groups based on the median value of the product, which was obtained by multiplying the expression level of these lncRNAs with the corresponding expression coefficients.

### 2.5. Cytoscape Analysis

The relationship between genes that were positively and negatively associated with smoking and lncRNAs was analyzed using Cytoscape (3.8.0) [21].

### 2.6. Immune Infiltration Analysis

The CIBERSORT algorithm was used to evaluate the composition of 22 different immune cells by analyzing the gene expression profile data obtained from the GEO database. Gene expression among these groups was analyzed using the ggplot2 and ggpubr R packages. The correlation between genes and immune cells was assessed using the ggplot2, ggpubr, and ggExtra packages.

### 2.7. Survival Analysis

The survival and survminer R packages were used to analyze the prognostic significance of positive and negative lncRNAs, respectively. The Kaplan-Meier method was used to plot survival curves for the high- and low-risk groups, whereas the log-rank test was used to evaluate the statistical significance between the groups. *p* values < 0.001 were considered to indicate statistical significance.

### 2.8. Receiver Operating Characteristic (ROC) Curve Analysis

We used the positive and negative lncRNAs to construct the theoretical risk model. To compare their accuracy in evaluating the effect of smoking, ROC curve analysis was conducted using the survival, survminer, and timeROC packages in R. The area under the ROC curve of the positive and negative correlation lncRNA model represents the prognosis evaluation of the model for patients with lung cancer who smoke. The larger the area under the curve, the more accurate the model. We found that the area under the curve increased gradually and exceeded 0.5 (i.e., the threshold for accurate prediction of the effect of smoking).

### 2.9. Cox Regression Analysis

The survival package in R was applied, and univariate and multivariate Cox regression analyses were used to screen out positive and negative lncRNAs closely associated with smoking-induced lung cancer prognosis to build the model. Univariate and multivariate prognostic analyses included factors such as age, sex, tumor-node-metastasis (TNM) stage, and the proposed risk score. Correlations between gene expression and risk were analyzed to understand the effects of age, sex, and other clinical factors on lung cancer. We compared these factors with the risk score to determine which one had the greater influence on the prognosis of lung cancer.

### 2.10. Gene Ontology (GO) and Kyoto Encyclopedia of Genes and Genomes (KEGG) Enrichment Analysis

The GO and KEGG enrichment of smoking-related genes was studied using the R packages colorspace, stringi, ggplot2, clusterProfiler, http://org.hs.eg.db, and enrichplot. We selected the filtered condition PValueFilter = 0.05 or the filtered condition QValueFilter = 1.

## 3. Results

### 3.1. Differentially Expressed Genes in Smoking-Induced Lung Cancer

**Figure 1:**
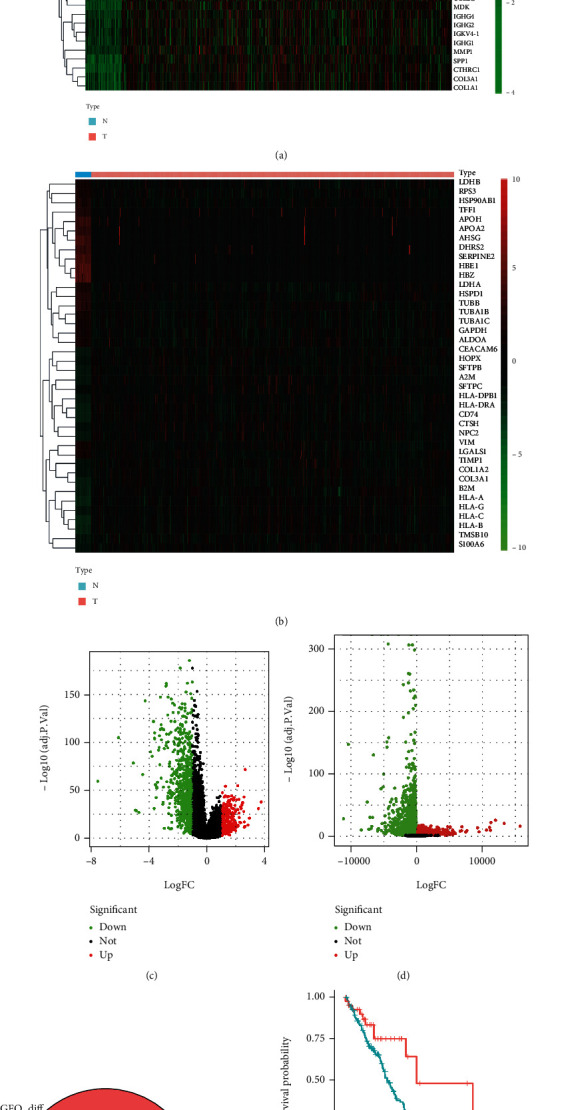
Differential expression of genes in lung cancer and effect of smoking on the prognosis of patients with lung cancer. (a) Genes obtained from The Cancer Genome Atlas (TCGA) dataset; blue denotes healthy subjects, pink represents patients with lung cancer, red represents positive association with high gene expression, and green represents negative association with low gene expression. The top 20 genes with the greatest positive and negative correlations were selected. (b) Genes obtained from the Gene Expression Omnibus (GEO) dataset, and others are similar to those in (a). (c) Volcano map of all differentially expressed genes identified through TCGA database; downregulated genes are indicated in green, and upregulated genes are indicated in red. (d) Differentially expressed genes identified in GEO. (e) The Venn diagram indicates the genes identified in TCGA and GEO databases. The differentially expressed genes are present in the intersection. Between the two databases, 562 differentially expressed genes were common. (f) Survival curve comparison for smoking and nonsmoking patients with lung cancer.

We compared genes expressed in lung cancer tissue samples with those from normal lung tissue samples from TCGA and GEO databases and identified 1,136 and 6,597 differentially expressed genes, respectively. The top 40 differentially expressed genes from each of the databases were selected to be represented in a heat map (Figures 1(a) and 1(b)). Among the 1,136 differentially expressed genes in TCGA, 786 were downregulated and 350 were upregulated (Figure 1(c)). Among the 6,597 differentially expressed genes in the GEO database, 3,177 were downregulated and 3420 were upregulated (Figure 1(d)). We intersected the differentially expressed genes in the two databases and obtained 562 common differentially expressed genes (Figure 1(e)). Additionally, we conducted a comparative study on the survival of smoking and nonsmoking patients with lung cancer and found that the death rate was significantly higher in smokers than in nonsmokers 4.5 years after tumor diagnosis (Figure 1(f), *p* < 0.05).

### 3.2. Genes Screened for Smoking History Association

**Figure 2:**
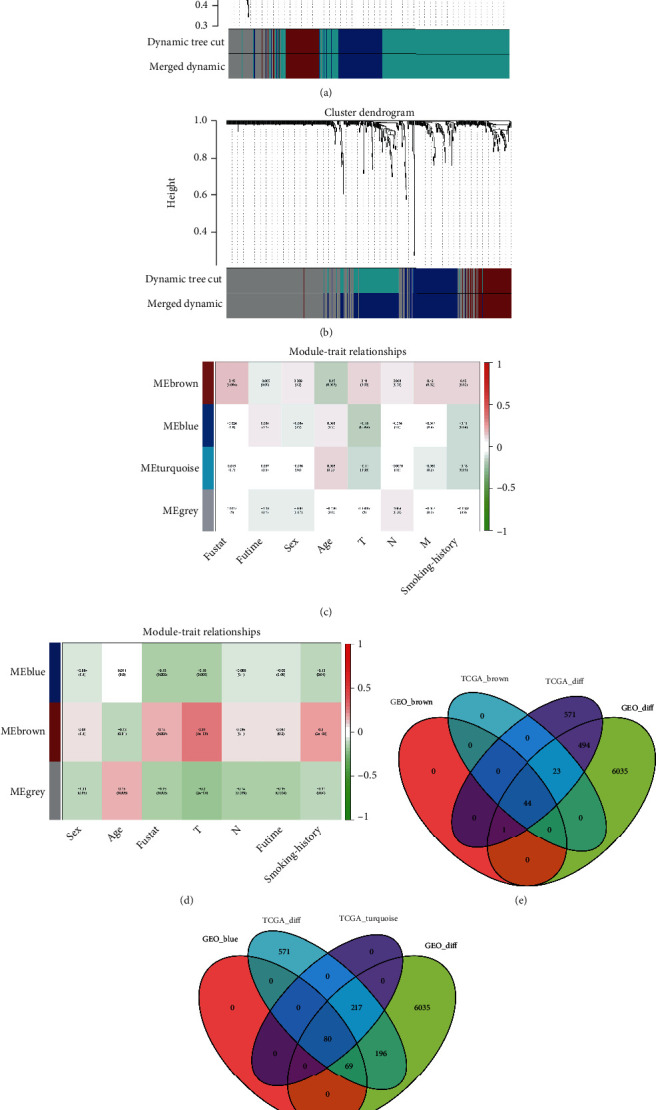
Effects of smoking on lung cancer. (a and b) The Cancer Genome Atlas (TCGA) and Gene Expression Omnibus (GEO) datasets; each tree fork represents a collection of genes for which the band is a series of modules. (c and d) Relationship between gene modules and traits in the GEO and TCGA datasets; pink represents positive and green denotes negative relation between traits and genes. The two databases contain 44 genes that are positively (e) and 80 genes that are negatively (f) associated with smoking.

We used WGCNA to conduct hierarchical cluster analysis on the 562 differentially expressed genes identified from the two databases and obtained a cluster tree diagram. By calculating when the threshold reaches a value of six, the constructed network can be made more consistent with the characteristics of a scale-free network (SFig. 1A, B). The genes in the lung cancer dataset were identified through hierarchical clustering based on the dissimilarity matrix, and a cluster tree was constructed (Figures 2(a) and 2(b)). The network module was set to contain at least 50 genes. The dynamic cutting method was used to identify different gene modules, and the modules with high similarity were combined. Finally, four and three different gene modules were obtained from TCGA (Figure 2(c)) and GEO (Figure 2(d)), respectively. The brown module in TCGA and GEO was positively correlated with smoking history, and 44 positively correlated genes were obtained by the intersection of the differential genes in the two databases and the genes in the brown module (Figure 2(e)). The turquoise module in TCGA and blue module in GEO were negatively correlated with smoking history. Eighty negatively correlated genes were obtained via the intersection with differential genes in the two databases (Figure 2(f)).

### 3.3. Function of Genes Associated with Smoking History

**Figure 3:**
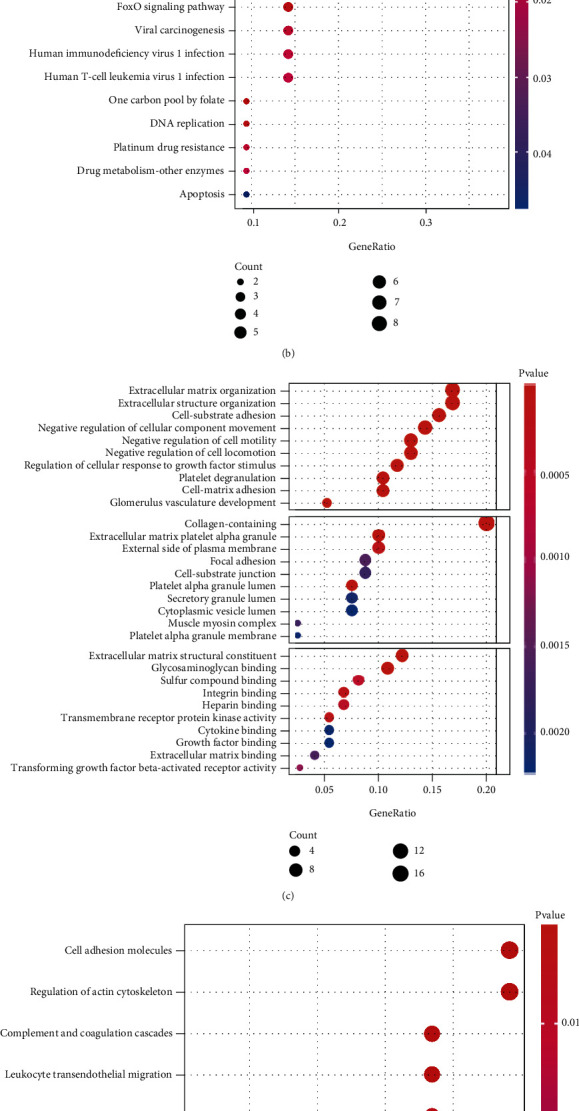
Gene Ontology (GO) analysis for functional enrichment of genes positively (a) and negatively (c) associated with smoking. Kyoto Encyclopedia of Genes and Genomes (KEGG) pathway enrichment analysis for determining the function of genes positively (b) and negatively (d) associated with smoking. (e) Cytoscape analysis showing the major functions of genes negatively associated with smoking.

Next, we analyzed the functions of the smoking-associated genes identified in the study. GO enrichment analysis of the positively correlated genes demonstrated that they are involved in nuclear division, organelle fission, and mitotic nuclear division (Figure 3(a)). KEGG pathway enrichment analysis revealed significant enrichment of these genes in the cell cycle, progesterone-mediated oocyte maturation, oocyte meiosis, and the p53 signaling pathway (Figure 3(b)). The GO enrichment analysis of negatively correlated genes revealed that they are associated with extracellular matrix organization, extracellular structure organization, cell-substrate adhesion, and adhesion accumulation (Figure 3(c)). In addition, KEGG enrichment analysis showed that these genes play a role in cell adhesion molecules, the regulation of actin cytoskeleton, complement and coagulation cascades, and leukocyte transferred migration. Interestingly, the negatively correlated genes were closely associated with the coronavirus disease (COVID-19) pathway (Figure 3(d)).

From the above functional enrichment results, we concluded that the genes negatively associated with smoking history are closely related to the functions of immunity and COVID-19. Next, we used Cytoscape to demonstrate the functions of negatively correlated genes (Figure 3(e)). These genes were closely associated with negative regulation of leukocyte apoptotic process, negative regulation of migration, and negative regulation of leukocyte immune processes such as the response to cytokine stimulus.

### 3.4. Relationship between Smoking and Immunity

**Figure 4:**
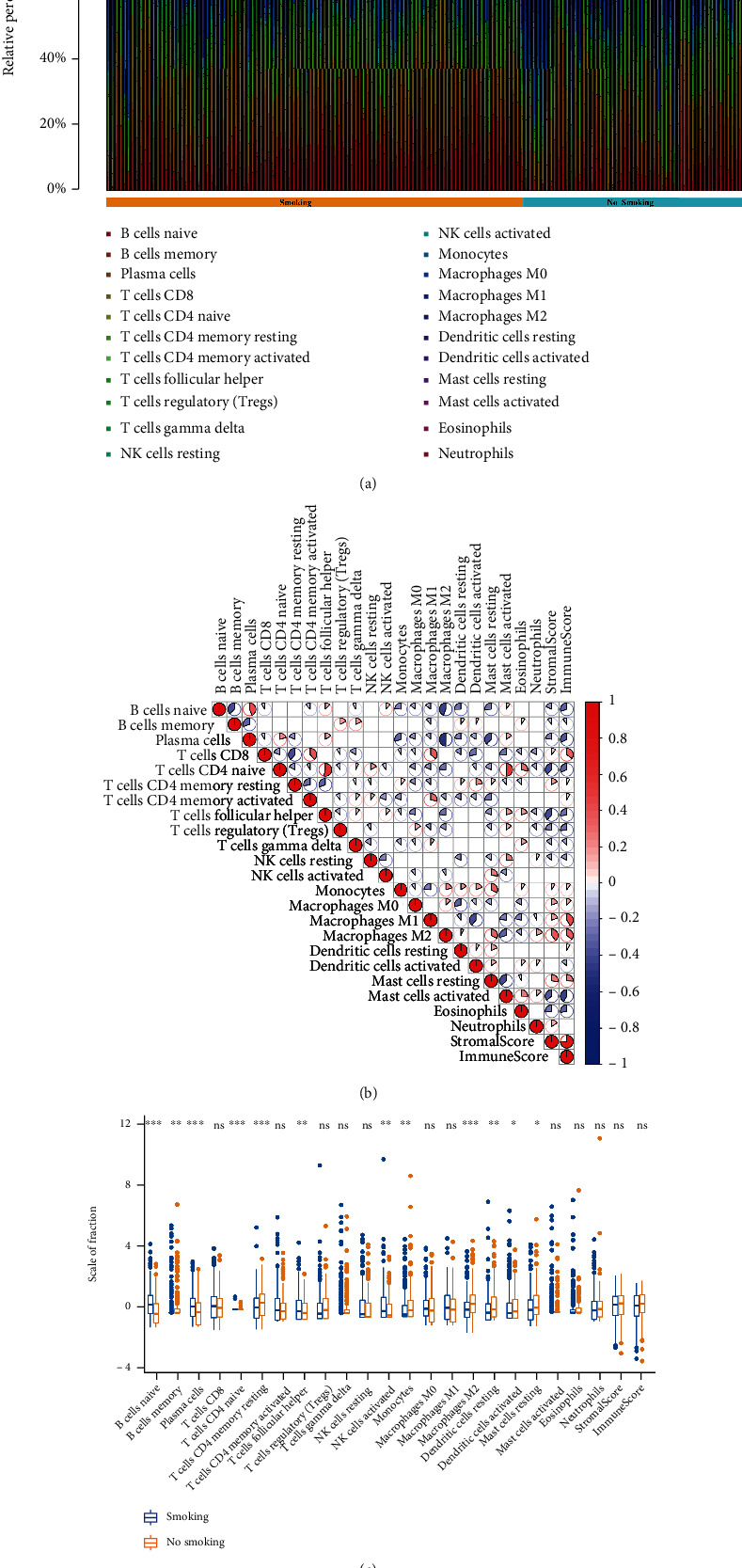
Effects of smoking on the immune microenvironment. (a) Infiltration rate of immune cells in the smoking and nonsmoking patient groups. (b) Relationship between different immune cell types. A pie diagram showing the positive and negative correlations in red and blue, respectively. (c) Relationship of different immune cell types observed in smoking and nonsmoking patients with lung cancer.

Based on the above KEGG enrichment analysis and Cytoscape results, genes negatively associated with smoking history were found to be closely related to immunity. Hence, we assessed immune cell infiltration in smoking and nonsmoking patients with lung cancer (Figure 4(a)). Naive CD4 T cells were most closely correlated with helper T cells and activated mast cells. Plasma cells and M2 macrophages exhibited the most negative correlation (Figure 4(b)). In addition, 12 immune cell types (naive and memory B cells, plasma cells, naive CD4 T cells, resting memory CD4 T cells, follicular helper T cells, activated natural killer (NK) cells, monocytes, M2 macrophages, resting and activated dendritic cells, and resting mast cells) were significantly different between the smoking and nonsmoking patients with lung cancer (Figure 4(c)).

### 3.5. Establishment of Smoking-Related lncRNA Models

**Figure 5:**
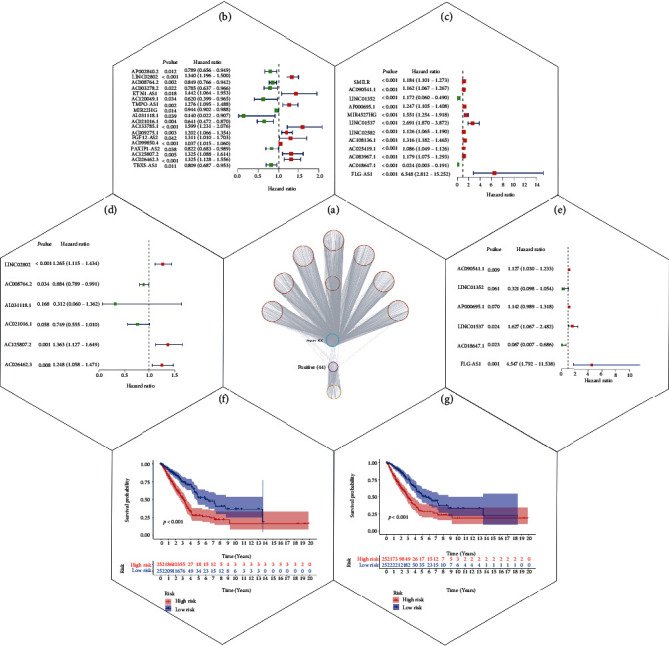
Construction of long noncoding (lnc) RNA models and analysis of their influence on smoking-induced lung cancer prognosis. (a) Cytoscape was used to associate the genes identified in the study with their subsequent expression of lncRNAs. Univariate Cox regression analysis was performed on the expression of lncRNAs for genes positively (b) and negatively (c) associated with smoking in The Cancer Genome Atlas (TCGA) and Gene Expression Omnibus (GEO) databases. Multivariate Cox regression analysis was performed on lncRNAs screened by a single factor positive (d) and negative (e) correlation in TCGA and GEO databases. Survival curves for high- and low-risk lncRNAs positively (f) and negatively (g) associated with smoking. *p* values < 0.001 were considered to indicate statistical significance.

We used Cytoscape to identify the corresponding lncRNAs of the genes that were positively and negatively associated with smoking, termed as positive and negative lncRNAs, respectively (Figure 5(a)). Univariate Cox analysis was used to obtain positive and negative lncRNAs that were closely associated with the prognosis of lung cancer (Figures 5(b) and 5(c)). Multivariate Cox analysis was used to identify positive and negative lncRNAs that could independently affect the prognosis of lung cancer (Figures 5(d) and 5(e)). Using multivariate Cox analysis, we identified six positive and six negative lncRNAs. Using these 12 lncRNAs for the models, the median of the sum of the product of the expression level of each lncRNA and the expression coefficient of each lncRNA were used to divide the samples into high- and low-risk groups (Figures 5(f) and 5(g)).

**Figure 6:**
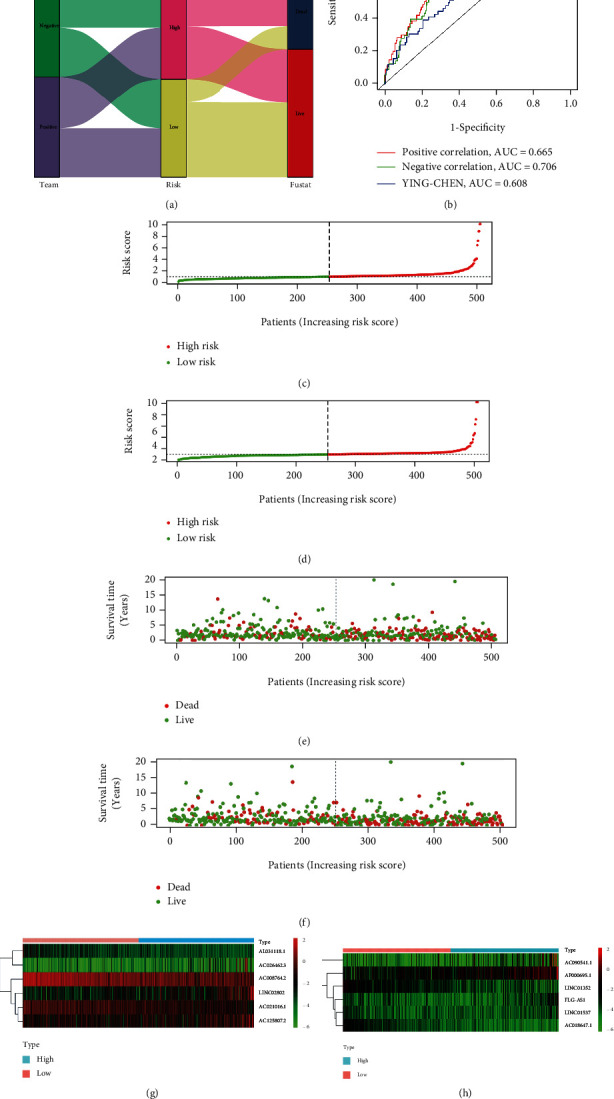
Comparison and risk assessment of the smoking-associated lung cancer prognostic models. (a) Sankey diagram. Half are high risk, while half are low risk. There was higher survival than death in the low-risk sample. (b) Receiver operating characteristic curve analysis for the prognostic accuracy of the model. (c and d) Patient risk scores for positive and negative long noncoding (lnc) RNAs. (e and f) Survival rates in the high- and low-risk patient groups for positive and negative lncRNAs. (g and h) Heat maps of gene expression in the risk model for the high- and low-risk groups with positive and negative lncRNAs.

Next, we compared the survival of patients in the high- and low-risk groups and found a significant increase in death in the high-risk group in both the positive and negative lncRNA models (Figure 6(a)). To further evaluate the accuracy of the two models for risk assessment of patients who smoked, we used the ROC curve. The area under the curve (AUC) value for negative lncRNA model was 0.706, the AUC value for positive lncRNA model was 0.665; the AUC value for negative lncRNA model was larger than the AUC value for positive lncRNA model; hence, negative lncRNAs were more accurate in the assessment of smoking-induced risk. We also compared our model with that of Chen et al. [19]. In their study, RXFP1, RAMP2-AS1, LINC00312, and LINC00472 were identified as key lncRNAs in smoking-associated lung cancer; however, their area under the curve (AUC) value is 0.608 and is smaller than that in our study, indicating that our model is more accurate (Figure 6(b)). The risk values of patients in the high- and low-risk groups were also calculated (Figures 6(c) and 6(d)). Notably, patients in the low-risk group had improved prognosis and survived longer than patients in the high-risk group (Figures 6(e) and 6(f)). Additionally, more patients survived in the low- than in the high-risk group. Finally, the expression of lncRNAs identified using the model in the high- and low-risk groups was visualized using a heat map (Figures 6(g) and 6(h)). Smoking was found to be positively correlated with certain lncRNAs (i.e., AL031118.1, AC026462.3, AC008764.2, LINC02802, AC021016.1, and AC125807.2) and negatively correlated with other lncRNAs (i.e., AC090541.1, AP000695.1, LINC01352, FLG-AS1, LINC01537, and AC018647.1).

### 3.6. Comparison of the Model with Other Clinical Traits and Identification of Pathways Associated with the Model

**Figure 7:**
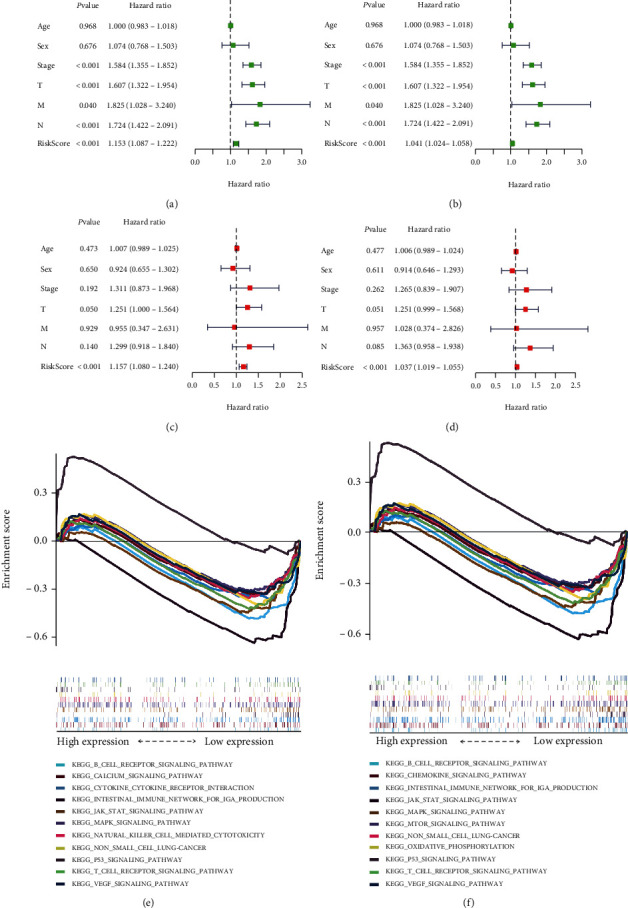
Relationship between the risk model and clinical factors. (a and b) Single factor prognostic analysis included age, sex, tumor-node-metastasis (TNM) stage, and the risk scores of patients with lung cancer with positive and negative long noncoding (lnc) RNAs. (c and d) Multifactor prognostic analysis included age, sex, TNM stage, and the risk scores of patients with lung cancer with positive and negative lncRNAs. (e and f) Pathways identified using the positive and negative lncRNA model.

We compared the prognostic significance of the positive and negative lncRNA models with that of other clinical traits. Univariate Cox analysis showed that both models, together with stage and TNM, could influence the prognosis of patients (Figures 7(a) and 7(b)). Multivariate Cox analysis showed that both models could be used as independent prognostic factors for patients (Figures 7(c) and 7(d)). The positive lncRNA model was associated with the B cell receptor signaling pathway, intestinal immune network for IgA production, the p53 signaling pathway, and other immunological and tumor-related pathways (Figure 7(e)). Similarly, the negative lncRNA model was associated with the B cell receptor signaling pathway, the chemokine signaling pathway, intestinal immune network for IgA production, the JAK-STAT signaling pathway, the MAPK signaling pathway, and other immunological and tumor-related pathways (Figure 7(f)).

## 4. Discussion

Smoking remains a leading risk factor for early death and disability worldwide [22]. In this study, we investigated the relationship between smoking and lung cancer in silico, hoping to further elucidate their relationship. Genetic changes play an important role in lung cancer [23]. Notably, gene expression changes have been reported in the lung cancer tissues of smokers and nonsmokers with lung adenocarcinoma [24]. In our study, we identified 562 differentially expressed genes from lung cancer and normal tissues from the TCGA and GEO databases. Two of the genes identified in our study were ROS1 [25, 26], which is widely studied in the treatment of lung cancer, and IL-6 [27], which plays a role in tumor microenvironment changes. To further elucidate the relationship between smoking and lung cancer, we analyzed the prognosis of smoking and nonsmoking patients with lung cancer. The 5-year survival rate of lung cancer patients has increased significantly with advances in early diagnosis and treatment [28]. However, continuing to smoke after the diagnosis of lung cancer is associated with higher therapeutic toxicity, risk of recurrence, and poor prognosis [29]. In our study, although no difference in survival was detected between the patient groups initially, differences were noted 4.5 years after the diagnosis of lung cancer. Hence, we concluded that there was a significant survival difference between the groups after 4.5 years.

Several genes were found to be associated with smoking. Using WGCNA, we divided the genes into those that were positively and negatively associated with smoking. This division was conducive to the subsequent study of gene function. Cigarette smoke contains various chemicals, and smoking-related diseases can occur through the effects on gene expression that occur via DNA methylation [30]. Through GO and KEGG enrichment analysis, we found that genes negatively associated with smoking were closely related to immunity. When chemicals in cigarettes enter the airway, they prompt macrophages to launch an inflammatory response for their removal [31]. Immune cells in the bronchus regulate the inflammatory response through DNA methylation, secretion regulation, and proinflammatory signaling molecules [32, 33]. Hence, smoking is closely related to a variety of immune diseases [34, 35].

We also used Cytoscape to analyze the function of genes inversely associated with smoking. We found that these genes were involved in the negative regulation of leukocyte apoptotic process, chemokine-mediated signaling pathway, and negative regulation of response to cytokine stimulus. da Silva D. et al. reported that the number of leukocytes and neutrophils increases in the airways of mice exposed to cigarette smoke [36]. Consistently, we noted a negative regulation of gene function in leukocyte apoptosis in smoking patients with lung cancer. Alshehri et al. demonstrated that smoking induces chemokine production, which affects airway inflammation by regulating interleukin- (IL-) 8 [37]. In our study, we found significant differences in CD4 T cell infiltration between smoking and nonsmoking patients. Previous studies on CD4 T cells in patients with a smoking habit have reported similar results. For example, Wasén et al. found that smoking induces the apoptosis of CD4^+^ T cells in patients with rheumatoid arthritis [38]. Furthermore, Wang and Guo found decreased infiltration of resting memory CD4 T cells and increased infiltration of follicular helper T cells in patients with lung squamous cell cancer who smoked or had quit smoking for less than 15 years [39]. The results of our study are consistent with those of these studies.

NK cells [40, 41], monocytes [42], macrophages [43], B cells [44], dendritic cells [45], and mast cells [46] have also been associated with smoking. In our study, we found significant differences in plasma cell infiltration between smoking and nonsmoking patients, which has rarely been reported. Thus, our study provides a new direction for further research: to determine the influence of smoking on the immune microenvironment. Notably, we identified that the genes negatively associated with smoking were closely related to the COVID-19 pathway. Thus, we propose that smoking can increase the possibility of respiratory tract infections; however, owing to the small number of cases in this study, there is no favorable evidence to support the relationship between COVID-19 and smoking [47]. In conclusion, this bioinformatic study will provide theoretical support for further research focused on clarifying the relationship between COVID-19 and smoking.

lncRNAs play an important role in a variety of diseases and a huge role in smoking-induced tumors [48]. We identified lncRNAs that were positively and negatively associated with smoking and constructed the smoking-associated lncRNA model through univariate and multivariate Cox analyses. Upon survival analysis, we found that the model is a faithfully prognostic factor of lung cancer. Currently, there are relatively few reports on the prediction of tumor prognosis based on lncRNAs. Gong et al. reported that LINC01537 plays an important role in energy metabolism, as well as in the prognosis of lung cancer [49]. This lncRNA was also identified using our model. However, there are few reports on the synergistic effect of lncRNAs on tumor prognosis and even fewer studies on the prognosis of lung cancer based on lncRNAs. We constructed a prognostic lncRNA model for lung cancer, evaluated the influence of the model on the risk of lung cancer, and performed comparisons with other clinical traits. We found that lncRNAs can effectively be considered as an independent risk factor for lung cancer. Thus, our study acts as a foundation for future research on the role of lncRNAs in lung tumor prediction.

## 5. Conclusions

Throughout our study, we screened out the smoking-related genes and analyzed their functions to better understand their role in the development of lung cancer. The discovery of a relationship between negatively associated genes and immunity would provide theoretical support for the study of the immune microenvironment in smoking-induced lung cancer. In addition, the current study focused on elucidating the role of smoking-related lncRNAs in lung cancer progression. The findings of the study revealed that smoking-related lncRNAs are a good prognostic marker of survival and risk in patients with lung cancer. The accuracy and advantages of our model were further highlighted by comparing it with the lncRNAs reported in the previous studies. However, this study still had some limitations. For example, the research was limited to the TCGA and GEO databases, and more databases and different population samples are needed for verification.

## Data Availability

The data that support the findings of this study were obtained from TCGA (https://portal.gdc.cancer.gov/) and GEO (gse68465) databases.

## Supplementary Materials

Supplementary Materials SFig. 1A and 1B: determination of soft-thresholding power in weighted gene coexpression network analysis (WGCNA) in (A) the TCGA dataset and (B) the GEO datasets.
