# Supplementary figures and images for: Role of Long Noncoding RNAs in Smoking-Induced Lung Cancer: An In Silico Study
Source: Comput Math Methods Med. 2022 Apr 30;2022:7169353. doi: 10.1155/2022/7169353 (PMC9070410; doi:10.1155/2022/7169353)

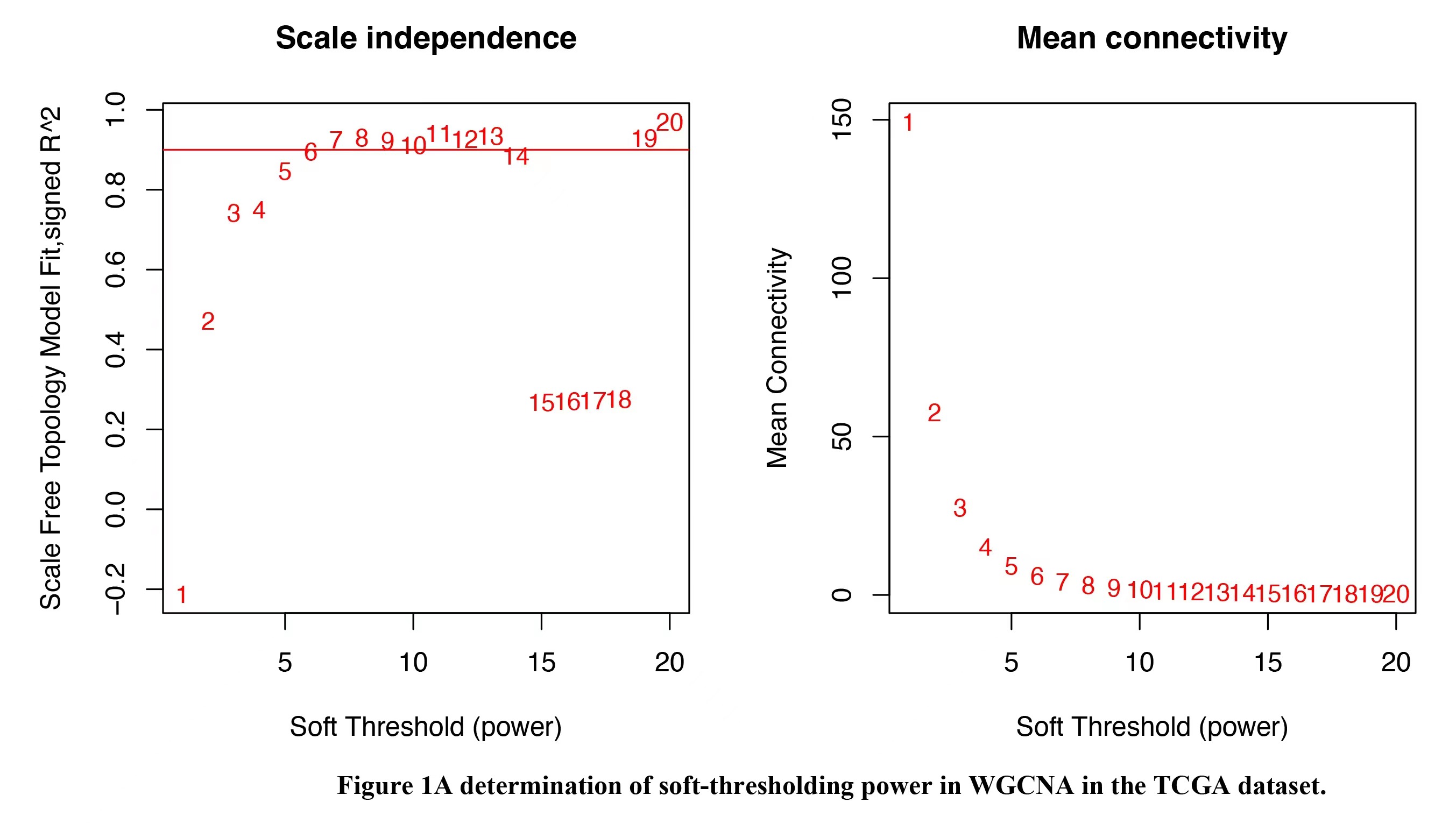

Supplement: Supplementary Materials — SFig. 1A and 1B: determination of soft-thresholding power in weighted gene coexpression network analysis (WGCNA) in (A) the TCGA dataset and (B) the GEO datasets. [file 7169353.f1.zip › 7169353.f1/SFigure1A (1).jpg]

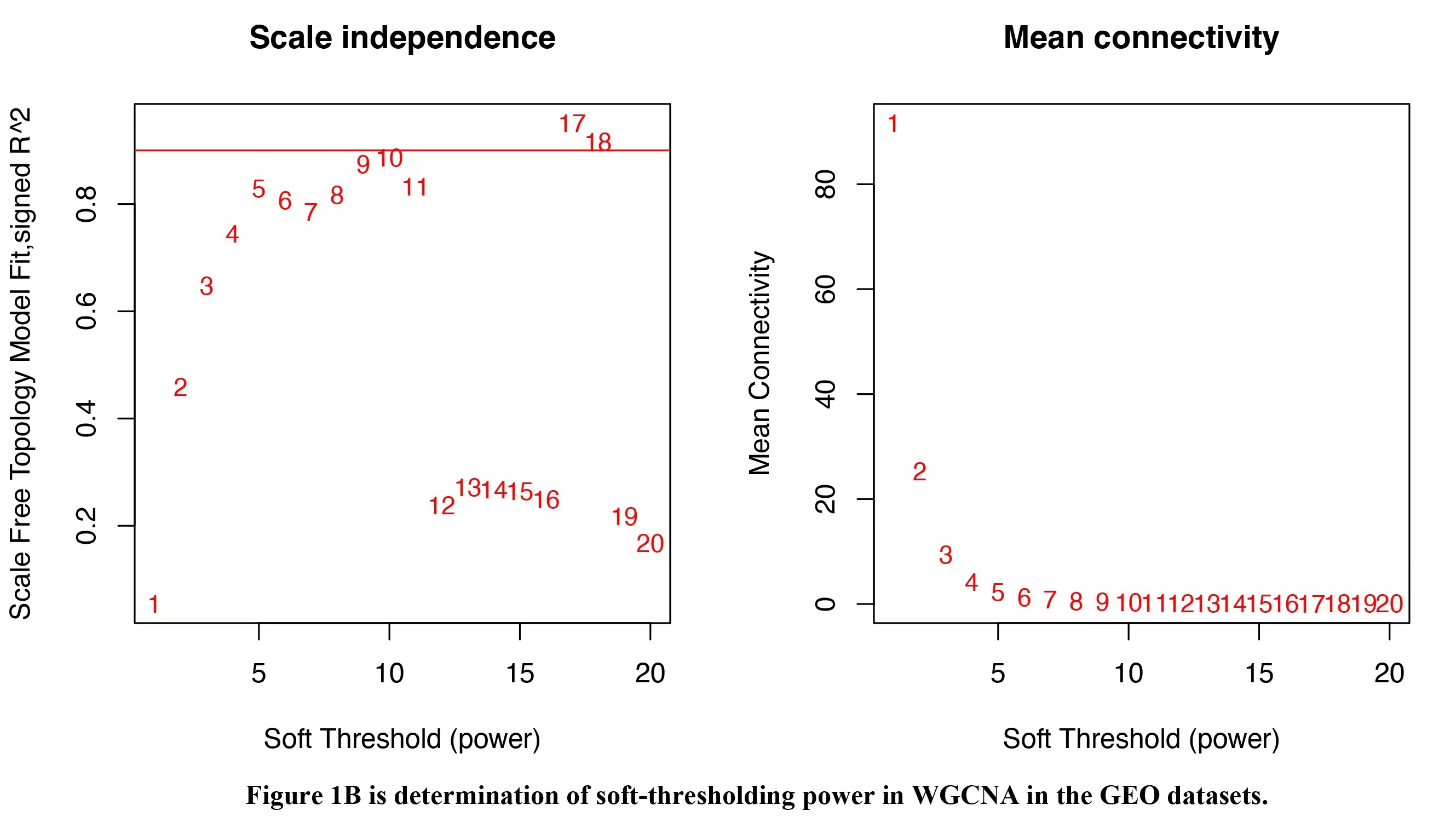

Supplement: Supplementary Materials — SFig. 1A and 1B: determination of soft-thresholding power in weighted gene coexpression network analysis (WGCNA) in (A) the TCGA dataset and (B) the GEO datasets. [file 7169353.f1.zip › 7169353.f1/SFigure1B (1).jpg]
